# Liver-bone crosstalk in non-alcoholic fatty liver disease: Clinical implications and underlying pathophysiology

**DOI:** 10.3389/fendo.2023.1161402

**Published:** 2023-03-10

**Authors:** Jiahui Zhao, Hongyan Lei, Tianyi Wang, Xuelian Xiong

**Affiliations:** Ministry of Education Key Laboratory of Metabolism and Molecular Medicine, Department of Endocrinology and Metabolism, Zhongshan Hospital, Fudan University, Shanghai, China

**Keywords:** non-alcoholic fatty liver disease, osteoporosis, secreted protein, osteoblasts, osteoclasts

## Abstract

Osteoporosis is a common complication of many types of chronic liver diseases (CLDs), such as cholestatic liver disease, viral hepatitis, and alcoholic liver disease. Non-alcoholic fatty liver disease (NAFLD) is a highly prevalent metabolic liver disease, affecting almost one third of adults around the world, and is emerging as the dominant cause of CLDs. Liver serves as a hub for nutrient and energy metabolism in the body, and its crosstalk with other tissues, such as adipose tissue, heart, and brain, has been well recognized. However, much less is known about the crosstalk that occurs between the liver and bone. Moreover, the mechanisms by which CLDs increase the risk for osteoporosis remain unclear. This review summarizes the latest research on the liver–bone axis and discusses the relationship between NAFLD and osteoporosis. We cover key signaling molecules secreted by liver, such as insulin-like growth factor-1 (IGF-1), fibroblast growth factor 21 (FGF21), insulin-like growth factor binding protein 1 (IGFBP1), fetuin-A, tumor necrosis factor-alpha (TNF-α), and osteopontin (OPN), and their relevance to the homeostasis of bone metabolism. Finally, we consider the disordered liver metabolism that occurs in patients with NAFLD and how this disrupts signaling to the bone, thereby perturbing the balance between osteoclasts and osteoblasts and leading to osteoporosis or hepatic osteodystrophy (HOD).

## 1 NAFLD and Osteoporosis, two possible linked pathologies

### 1.1 NAFLD

**Figure 1:**
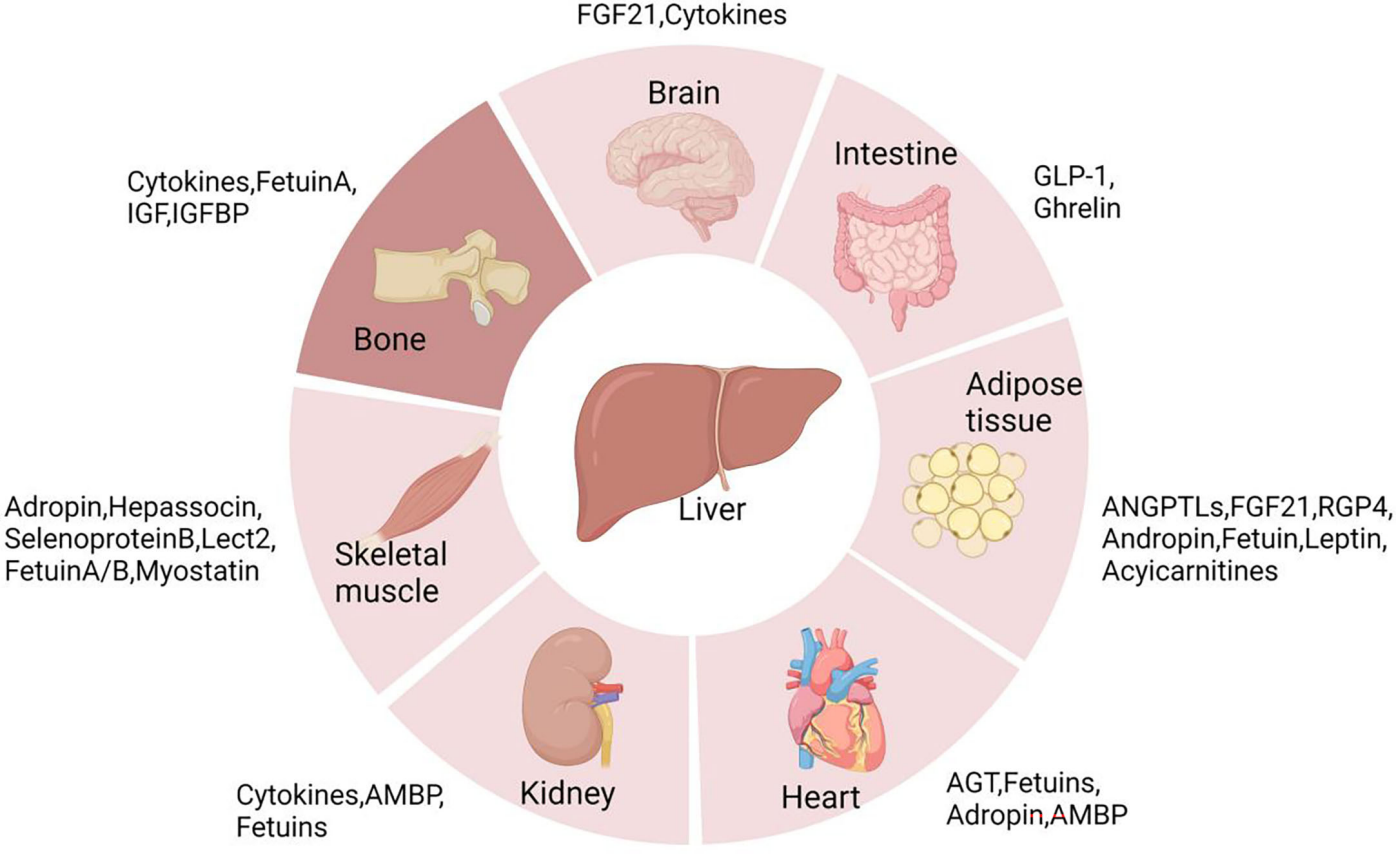
Communication between liver and distant organs. Liver is the metabolic center of the body. Because it is distant from many organs, liver usually secretes a variety of signaling molecules like hepatokines, cytokines, and enzymes into the circulation to modulate the function and metabolism of other organs.

Liver is the center of metabolism for the whole body, and liver communicates with other tissues *via* secreted hormones and metabolites (**Figure 1**). Over the past 10–20 years, non-alcoholic fatty liver disease (NAFLD) has become one of the most common causes of chronic liver diseases (CLDs) in Western countries, and the incidence is increasing steadily. However, it is noteworthy that the incidence of NAFLD is also rapidly increasing in Asia, especially in China. Owing to the development and long-term application of effective prevention and control measures, the morbidity of viral hepatitis has deceased consistently, and NAFLD has thus emerged as the most common etiology of CLD (1). Without intervention, it is expected that China will soon have the world’s highest number of patients with NAFLD (2).

NAFLD is characterized by the accumulation of lipid in hepatocytes, therefore, NAFLD has been considered the manifestation of metabolic syndrome in the liver. Meanwhile, it is also closely related to systemic metabolic disorder. NAFLD increases the risk of many different diseases. It is possible for NAFLD to progress to non-alcoholic steatohepatitis (NASH) or even to hepatocellular carcinoma (HCC). In cases of NAFLD, the metabolic functions of liver are markedly affected, and inflammatory cytokines secreted by liver increase (3). In this regard, the pathophysiology of NAFLD is similar to other CLDs, underscoring potential clinical significance of the relationships between NAFLD and osteoporosis and the importance of elucidating the underlying mechanisms of action in pathophysiology.

### 1.2 Normal bone metabolism

The key processes during bone metabolism are bone resorption and bone formation. Maintaining a proper balance between these two processes is critical for healthy bone growth and the maintenance of normal bone mass. Bone resorption is mediated by osteoclasts, which are multinucleated cells derived from circulating monocytes. Bone formation is mediated by osteoblasts that originate from mesenchymal cells. Osteoblasts replace bone after resorption and mineralize osteoid seams (4). Hence, precise coordination of osteoclast and osteoblast activity is required for maintaining osteohomeostasis, and disruption of this balance can manifest in diseases of the bone.

**Figure 2:**
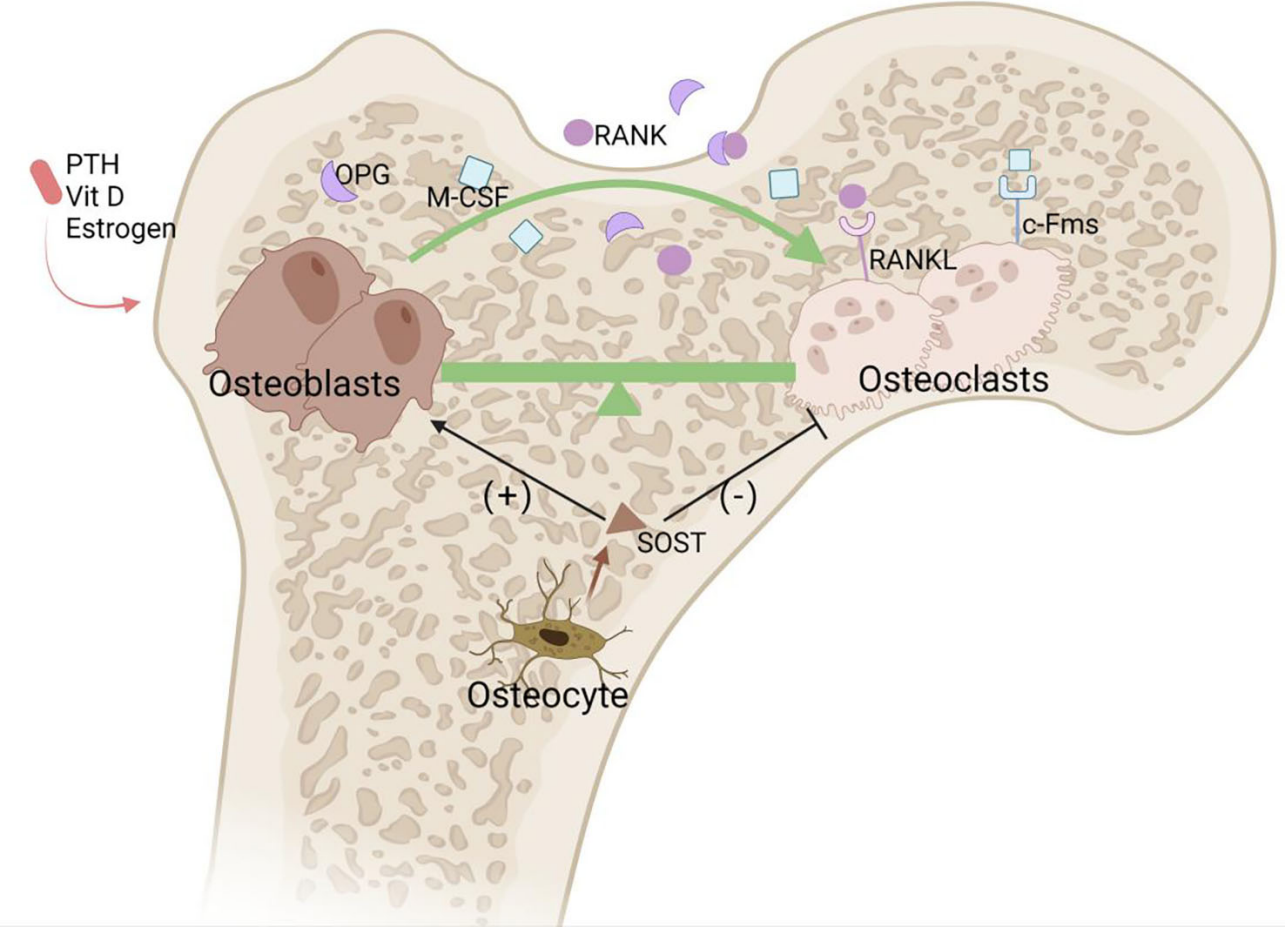
Osteoclasts and osteoblasts balance in bone remodeling. Bone remodeling is an orderly and coupled process of bone resorption and bone formation mediated respectively by osteoclasts and osteoblasts. RNAKL/RANK and M-CSF/c-Fms are the two most important pathways governing osteoclast proliferation and differentiation. OPG can bind to RANKL, inhibiting the interaction between RANKL and RANK. Estrogen, vitamin D, and small doses of PTH from the circulation can promote bone formation. Osteocytes also secrete SOST, which negatively regulates osteoblasts and positively regulates osteoclasts.

Osteoblasts secrete many factors, such as receptor activator of NF-κB ligand (RANKL), macrophage colony-stimulating factor (M-CSF), and osteoprotegerin (OPG), to regulate the functions of osteoclasts (5). RANKL is widely considered to be a key osteoclastic cytokine regulating bone turnover by binding the RANK receptor on the surface of osteoclast precursors to induce them to differentiate into mature osteoclasts; indeed, the RANKL–RANK axis is critical for the differentiation and maturation of osteoclasts. Osteoblasts also secrete OPG, which can block the binding of RANK to RANKL, suppressing bone resorption. Besides, M-CSF secreted by osteoblasts can positively regulate osteoclast activity. Osteocytes in the bone matrix can produce sclerostin (SOST), which increases osteoclast activity and decreases osteoblast activity (6)(**Figure 2**).

### 1.3 Osteoporosis

Osteoporosis is a public health issue that affects more than 200 million people worldwide (7, 8). A cross-sectional study of 20416 individuals in China showed that the prevalence of osteoporosis among those aged 40 years or older was 5.0% among men and 20.6% among women (9). Considering that the incidence of osteoporosis increases rapidly with age, it has become a major focus for researchers and clinicians. Osteoporosis is characterized by low bone mass and microstructural destruction, which increase bone fragility (7, 10). Bone fractures, particularly in the spine or hip, are the most serious complications of osteoporosis and contribute to significant morbidity and mortality (11). Primary osteoporosis is associated with the process of aging and accounts for 60% of all cases of osteoporosis. Of the more than 200 million individuals with osteoporosis, about 40% will have osteoporosis that secondary to some chronic conditions such as CLDs (11, 12). The specific mechanism by which CLDs cause osteoporosis has not been established. It is generally thought that primary cholestatic liver disease, viral hepatitis, and alcoholic liver disease are directly related to osteoporosis, and the possible link between these two diseases might be the inflammatory liver environment. In the circumstance of liver inflammation and fibrosis, hepatic stellate cells are activated and release oncofetal fibronectin, which acts on osteoblasts to decrease bone formation (12, 13). Liver also secretes peripheral CSF1 and inflammatory cytokine-like tumor necrosis factor (TNF) which respectively bind the colony-stimulating factor-1 receptor (c-Fms) and TNF receptor on osteoclast precursors, thereby increasing bone resorption (14, 15). The correlation between NAFLD and osteoporosis remains controversial: whereas some researchers have concluded that there is no correlation, others provided evidence that supports a correlation between NAFLD and osteoporosis (16, 17). Several studies have demonstrated that hepatic fibrosis may be more closely related to osteoporosis than simple steatosis (18–21). However, the clinical trials that have investigated the link between NAFLD and osteoporosis have many limitations, including: 1) inconsistence of inclusion criteria in different trial; 2) a large number of cross-sectional studies that could not prove causality; 3) difficulty applying the gold standard diagnostic method—liver biopsy—to diagnose NAFLD; and 4) other confounding factors, such as comorbidities and medication.

### 1.4 HOD

Almost all patients with CLDs experience changes in bone metabolism, with up to 75% of which showing clinical features of osteoporosis. Because of its high prevalence, there is a name for this syndrome—hepatic osteodystrophy (HOD)—which describes hepatic dysfunction-related changes in bone metabolism, such as decreased bone mineral density and deterioration of bone structure. If no remedial measures are taken, patients with HOD are at high risk for bone fractures that markedly affect quality of life and long-term prognosis and increase the death rate (22).

## 2 Interorgan crosstalk between the liver and bone

The physical distance separating liver and bone prevents direct physical interaction between these two tissues. Rather, liver must communicate with bone by secreting signaling molecules, such as proteins, enzymes, and cytokines, that circulate and affect bone function through endocrine signaling. Understanding the nature of endocrine signaling between liver and bone may enhance the prevention, diagnosis, and treatment of CLD-caused bone metabolic diseases, such as osteoporosis or HOD (23). Here we summarize recent studies on liver-bone hormonal signaling in bone homeostasis and disease.

### 2.1 IGF-1

Insulin-like growth factor-1 (IGF-1) is a key growth factor secreted by liver. It is also an endocrine hormone that is induced by growth hormone (GH). The structure and function of IGF-1 are similar to insulin, and IGF-1 can promote growth by enhancing absorption of amino acids, glucose, and fatty acids.

#### 2.1.1 Circulating and local IGF-1 play an important role in bone metabolism

**Figure 3:**
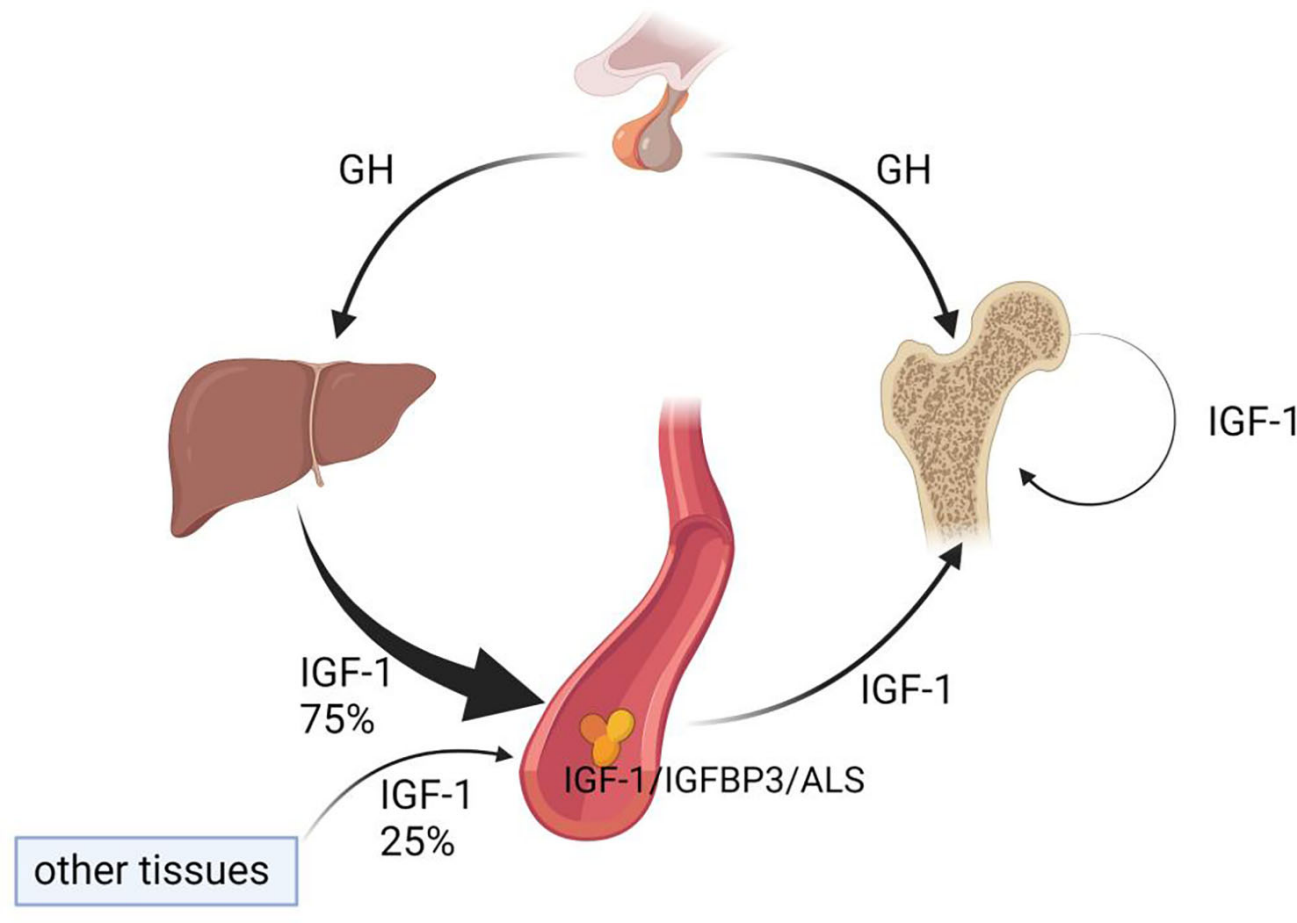
Circulating and local IGF-1 both positively effect bone remodeling. Seventy-five percent of IGF-1 in the circulation is liver derived, whereas twenty-five percent of IGF-1 originates from other tissues. Bone itself can secrete a small amount of IGF-1; although the quantity is relatively small, this locally produced IGF-1 plays an important role in bone remodeling.

Liver is the primary source of circulating IGF-1, but it is not the only source. Other tissues and organs, such as bone, secrete limited amounts of IGF-1 locally (24). IGF-1 binds with IGF binding protein 3 (IGFBP3) and Acid-labile subunit (ALS) to form a ternary complex, which is the main storage form for IGF-1 that can considerably extend its half-life (25, 26). In 1998, Cemborain et al. reported that bone mass was decreased following CCl4 treatment in rodents with experimental liver cirrhosis. Although IGF-1 is mainly induced by GH in the liver, they are not completely dependent on each other for the function of promoting body growth (27). In fact, liver and bone damages caused by chronic CCl4 intoxication is reminiscent of the human pathology of HOD (28), which is consistent with a relationship between NAFLD and HOD. A study comparing various growth indicators of *Ghr^+/−^* mutant, *Igf1^+/−^* mutant, and *Ghr^+/−^/Igf1^+/−^* double-mutant mice, showed that GH and IGF-1 promote growth through both independent and dependent functions. Although circulating IGF-1 decreases without GH, local IGF-1 production is still active (29). This implies that local IGF-1 production may play a broader role in growth promotion than is currently appreciated. Another study found that, in the circulation of liver-specific *Igf-1* knockout mice and ALS-specific knockout mice, IGF-1 decreased by 75% and 65%, respectively, but those mice still displayed relatively normal growth and development (25). In addition, in liver *Igf-1* and ALS double-knockout mice, circulating IGF-1 decreased further, and bone growth was substantially attenuated. These findings suggest that a threshold concentration of circulating IGF-1 (10%~25%) is necessary for normal bone growth, and that the IGF-1/IGFBP3/ALS ternary complex plays an important role in osteoporosis pathophysiology (**Figure 3**). Yakar et al. showed that locally produced IGF-1 in bone played a more dominant role than circulating IGF-1 in maintaining bone integrity. However, when local IGF-1 production was blocked, circulating IGF-1 demonstrated a compensatory effect (30). In fact, it has been demonstrated that both circulating IGF-1 and local IGF-1 participate in bone growth through different mechanisms (31). Moreover, GH and IGF-1 are important regulators of bone homeostasis and longitudinal bone growth before and during adolescence and also play a crucial role in maintaining bone mass during adulthood (29).

#### 2.1.2 Decreased IGF-1 levels in patients with osteoporosis

In 1995, Ravn et al. measured serum IGF-1 concentration in 107 healthy females and 116 females with osteoporosis. They found that the level of serum IGF-1 in females with osteoporosis was 30% lower than healthy females (32). A few years later, Kurland et al. found that, in 14 male patients with idiopathic osteoporosis, 11 had significantly reduced serum IGF-1 levels (33). Thus, decreased IGF-1 levels have been observed in both male and female patients with osteoporosis.

#### 2.1.3 Decreased levels of IGF-1 in patients with NAFLD

Normally, IGF-1 levels rapidly increase in adolescence and then steadily decease with age. However, a cross-sectional study of 168 obese children and adolescents with or without NAFLD found that IGF-1 levels of the NAFLD group were lower than IGF-1 levels of the control group (34). Such a negative relationship is found not only in young people but also in other age groups. Another cross-sectional study of 142 patients found that low serum IGF-1 levels were associated with increased histological severity of NAFLD after strict control for age, body mass index (BMI), and many other confounding factors (35). A meta-analysis of clinical trial data give the same result: IGF-1 levels were lower in patients with NAFLD compared to healthy controls (36). Therefore, IGF-1 may be used as a potential biomarker for NAFLD.

#### 2.1.4 IGF-1 is a potential therapeutic target

It has been proposed that low doses of IGF-1 constitute an alternative therapy that normalizes IGF-1 serum levels, thereby improving the expression of most proteins closely related with bone formation and reducing bone resorption (37). However, IGF-1 has a wide range of physiological effects as well as poor penetrance in the bone. For example, IGF-1 can stimulate the growth of internal organs, especially kidney and spleen. To address these obstacles for IGF-1 therapy, Lui et al. developed a cartilage-targeting single-stranded human antibody fragment (CaAb) that targets cartilage to deliver therapeutic molecules to growth plates, thereby increasing therapeutic efficacy while minimizing adverse effects on other tissues. Subcutaneous injection of CaAb–IGF-1 fusion protein increased overall growth plate height without increasing the proliferation of renal cortical cells (38).

At present, an IGF-1 receptor monoclonal antibody, Tepezza, is the first and only drug approved by the U.S. Food & Drug Administration for the treatment of thyroid eye disease (TED). Tepezza can markedly improve exophthalmos and diplopia symptoms in different subgroups of patients with TED, and most patients achieve long-term remission (39). However, no studies have been conducted to determine whether this drug has any beneficial effects on bone metabolism—something that may be apparent during long-term use.

### 2.2 FGF21–IGFBP1 axis

Fibroblast growth factor 21 (FGF21) is a protein secreted primarily by the liver, but it can also be expressed in white adipose tissue (WAT), pancreas, adipocytes, and skeletal muscle in response to physiological stimuli and/or pathological conditions (40, 41). FGF21 is primarily involved in the regulation of cell proliferation, growth and differentiation, and cell metabolism, but it is also an important endocrine regulator of glycolipid metabolism, playing an important role in enhancing β-oxidation, ketogenesis, gluconeogenesis, browning of WAT, and insulin synthesis in pancreas. It is generally thought that FGF21 could be a potential therapeutic agent for metabolic diseases, such as diabetes (including NASH), and synthetic recombinant human FGF21 (rhFGF21) has now entered clinical trials. Whether rhFGF21 could lead to osteoporosis needs to be addressed. Clinical tiral data revealed that FGF21 analog could lead to osteoporosis complications (42). Recently, other studies have found that FGF21 promotes IGFBP1 expression and also boosts bone resorption (43).

#### 2.2.1 Increased serum FGF21 levels in patients with NAFLD/NASH

It was shown in a prospective study that serum FGF21 levels were increased in patients with NAFLD/NASH, and this study was also the first to find that FGF21 mRNA expression was significantly increased in human liver with NAFLD (44). It is thought that elevated serum FGF21 levels in obese people reflect the existence of fatty liver, and FGF21 may be a biomarker of hepatic lipid accumulation in obesity (45). Another study measured FGF21 levels by ELISA in 82 patients with biopsy-confirmed NAFLD and in 77 control subjects and subsequently analyzed the association between FGF21 and NAFLD patient characteristics using multiple linear regression analysis. Serum FGF21 levels were elevated in patients with NAFLD independent of potential confounders and represented an independent predictor of hepatic steatosis (46). Thus, it is likely that elevated FGF21 in patients with NAFLD/NASH may serve as a protective mechanism.

#### 2.2.2 The FGF21–IGFBP1 signaling axis promotes bone loss

FGF21 has been widely studied for its beneficial effect on improving glucose and lipid metabolism. Interestingly, Wei et al. showed that FGF21 is a negative regulator of bone turnover. FGF21 forms a feed-forward loop to mediate and enhance the activity of peroxisome proliferator-activated receptor γ (PPAR-γ), thereby inhibiting formation of osteoblasts and stimulating adipogenesis in bone marrow mesenchymal stem cells. Importantly, this suggest that, despite the fact that FGF21 is beneficial for insulin resistance and type 2 diabetes, long-term use of FGF21 may lead to increased bone fragility (47).

It has been demonstrated that the hepatokine IGFBP1 can promote osteoclast production and bone resorption and is also an important mediator of FGF21-induced bone loss. The main function of IGFBPs is to form protein complexes with IGF to regulate its circulating levels, half-life, distribution in tissues, and binding of IGF to its receptors. In addition, IGFBP has some independent functions, including regulating transcription, cell migration, and apoptosis, which have received considerable attention in tumor and kidney disease. Wang et al. found that FGF21 induces the expression and secretion of IGFBP1 in liver, and IGFBP1 secreted by liver acts as an endocrine hormone by binding to receptor integrin β1 on osteoclast precursors, thereby enhancing RANKL signaling and osteoclast differentiation (48).

A search in PubMed found no relevant information about the levels of FGF21 and IGFBP1 in patients with osteoporosis, and future studies are needed to determine whether either or both of these proteins may be differentially expressed by patients with osteoporosis.

#### 2.2.3 IGFBP-1 is a possible therapeutic target

The metabolic effects of FGF21 have been exploited therapeutically, with rhFGF21 in clinical studies (49). However, the problem of osteoporosis caused by rhFGF21 treatment has not been resolved (50). It has been proposed that blocking IGFBP1 could prevent bone loss while preserving the insulin-sensitizing benefits of FGF21 therapy in patients with diabetes (48). In the future, IGFBP1 antagonists are expected to be tested for the potential to decrease bone loss and alleviate osteoporosis.

### 2.3 LCAT

Lecithin cholesterol acyltransferase (LCAT) is an enzyme synthesized and secreted by liver and mainly circulates as free protein or bound to lipoproteins. LCAT can convert free cholesterol on the surface of lipoproteins into cholesterol esters (CEs), which are essential for normal maturation, mutual conversion, and rearrangement of lipoproteins. The CEs produced by LCAT are stored in the core of high-density lipoprotein (HDL) particles, gradually increasing the size of HDL particles and causing HDL particles to mature, and eventually the cholesterol is sent to the liver (50, 51). LCAT-mediated cholesterol esterification is a rate-limiting step in the reverse transport of cholesterol and is considered a potential target for the prevention of atherosclerosis (52).

#### 2.3.1 HDL inhibits osteoclast production and induces osteoclast apoptosis

We have not identified any relevant research documenting changes in LCAT in patients with NAFLD and osteoporosis, but studies in mouse models have shown that dysfunctional and/or disordered HDL can affect bone mass in several different ways. Preclinical experiments in rodent models mostly show that HDL content is positively correlated with bone mass (53, 54), and clinical trials in postmenopausal women have reached a similar conclusion (55, 56). It has been shown that HDL is closely related to bone metabolism and that HDL can transport cholesterol from extrahepatic tissues to the liver for further metabolism (57). In bone, cholesterol forms an important part of the lipid raft, which is involved in signal transduction during osteoclast formation (58). In addition, the outflow of cholesterol from osteoclasts to HDL plays an important role in osteoclast apoptosis and fusion. HDL has been shown to inhibit osteoclast production and to induce osteoclast apoptosis (59).

#### 2.3.2 The relationship between LCAT and bone

**Figure 4:**
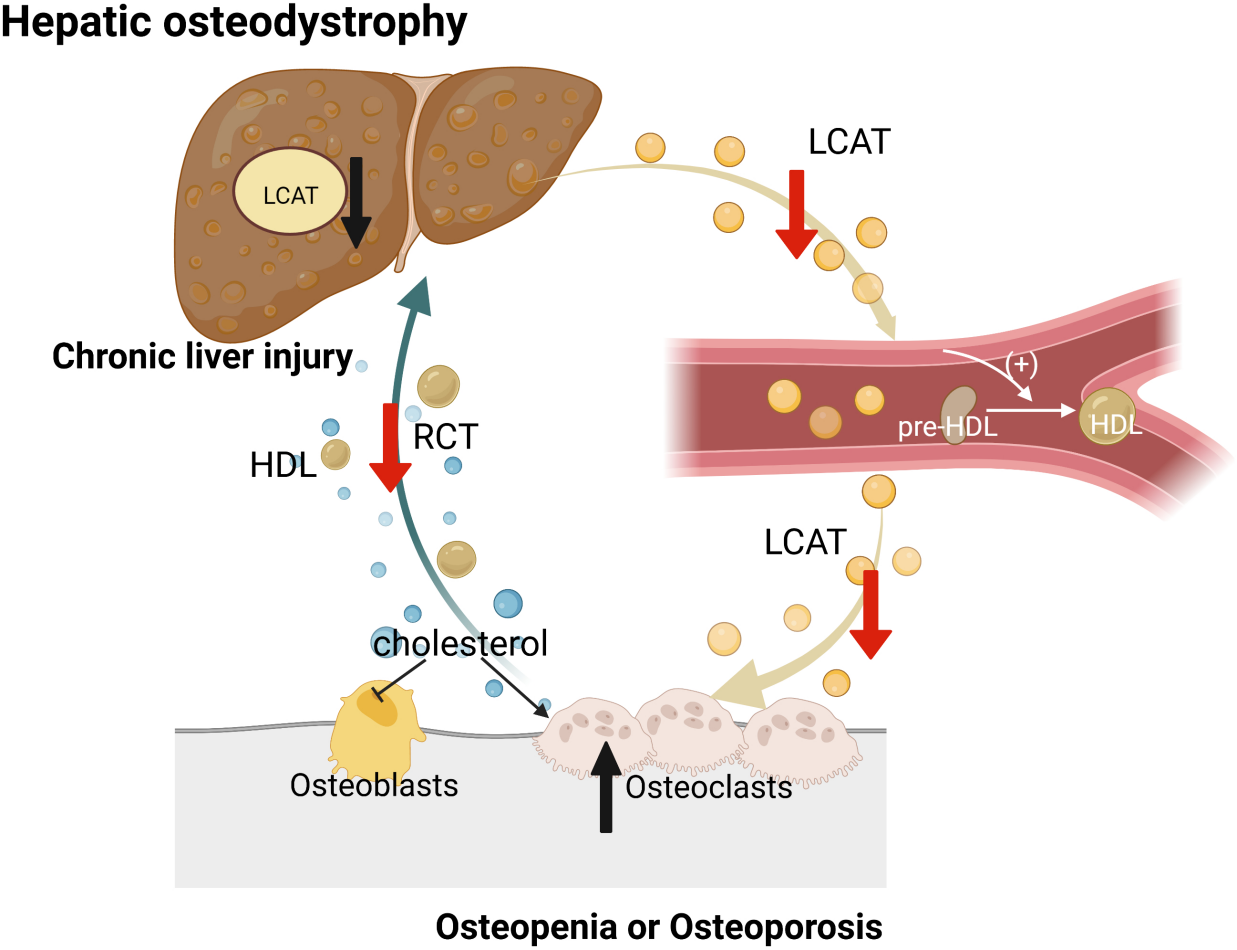
LCAT ameliorates bone loss by mediating RCT. Normally, liver secretes LCAT, which converts cholesterol obtained as HDL from surrounding tissues into cholesterol esters that are transported to liver for further metabolism. However, in the context of CLD, liver secretion of LCAT is blocked, resulting in restriction of RCT and accumulation of cholesterol in the bone that promotes osteoclast formation and inhibits osteoblast production.

LCAT is a key molecule for HDL maturation and reverse cholesterol transport, and its relationship with bone metabolism was recently described (53). A study of *Lcat^−/−^* mice showed that they are sensitive to the development of diet-induced obesity, especially to liver lipid deposition associated with NAFLD, and that obese *Lcat^−/−^* mice are more likely to develop osteoarthritis. Further, Lu et al. reported that, when chronic liver injury was induced during the pathogenesis of HOD, liver protein phosphatase 2ACα (PP2ACα) was upregulated, which led to a decrease in the expression of LCAT by liver as well as a defect in the reverse transport of cholesterol from bone to liver (**Figure 4**). The loss of LCAT function significantly aggravated bone loss related to HOD in mice. In addition, studies have shown that LCAT improves liver function in mouse models of HOD and alleviates liver fibrosis by promoting reverse cholesterol transport (RCT) from bone to liver (22).

#### 2.3.3 Potential therapeutic benefits of LCAT

Most data from preclinical studies suggest that increasing LCAT stimulates RCT and reduces atherosclerosis. Based on the fact that recombinant human LCAT (rhLCAT) can improve lipid metabolism, rhLCAT has been developed mainly for the treatment of cardiovascular diseases, such as atherosclerosis, and has entered phase II clinical trials with promising results from earlier phase studies (60). In addition, an activator of LCAT, synthetic apolipoprotein (apo)C-I, is being studied as a potential therapeutic for cardiovascular indications. If it is determined that rhLCAT, synthetic APOC-I, or other drugs to increase LCAT activity can also improve bone metabolism, their clinical applications will be much broader, and they could be prescribed to regulate lipid metabolism to improve obesity and obesity-induced cardiovascular disease, metabolic syndrome, and even diabetes, as well as to prevent related bone complications (61).

### 2.4 OPN

Osteopontin (OPN) is a phosphorylated glycoprotein secreted mainly by osteoblasts and osteoclasts that was originally found in the bone marrow. It has since been reported that immune cells can secrete OPN as well (62), and OPN has also been detected in other organs, such as liver. OPN concentration is high in breast milk and is an important protein for immune activity, which is beneficial for infant development and also improves infant immunity (63, 64). In healthy humans, OPN expression is low, but it can be stimulated to be secreted in large quantities and can participate in the immune response (65, 66), resisting mechanical stress, promoting fibrosis (67), and accelerating bone resorption, but the specific mechanisms of these functions remain to be fully elucidated (68).

#### 2.4.1 Increased OPN expression in NAFLD/NASH

In a clinical study, it was shown that patients with NAFLD had significantly higher serum OPN levels compared to the control group (69). Also, in a mouse model of NAFLD induced by a high-fat diet (HFD)/methionine–choline-deficient (MCD) diet, expression of OPN was increased in the liver of NAFLD mice, and silencing OPN improved liver lipid accumulation (70). In fact, OPN plays a role in the development of inflammatory chronic liver disease: liver lipid-associated macrophages (LAMs) express OPN, and OPN can activate hepatic stellate cells, promote liver fibrosis, and accelerate the development of NAFLD to NASH and even to HCC (71). Thus, OPN can be used as a biomarker for patients with NASH (72, 73).

#### 2.4.2 OPN is essential for bone metabolism

OPN can influence bone remodeling to regulate bone mass (74), and many studies have found OPN to be associated with the occurrence of various bone diseases. Yoshitake et al. constructed an OPN-knockout mouse model and found that, after oophorectomy, the bone volume decline in knockout mice was much lower compared to wild-type (WT) mice (75), indicating that OPN deletion can resist estrogen-deficiency-induced bone loss. Over a decade later, it was shown that the number of tartrate-resistant acid phosphatase(TRAP) positive cells in WT bone increased significantly after parathyroid hormone (PTH) treatment (76). However, no significant changes were observed in OPN-deficient bone. OPN deletion can resist PTH-induced bone resorption and is achieved by inhibiting the increase of osteoclasts. Clinically, some investigators have found that elevated serum OPN content is a risk factor for osteoporosis for postmenopausal women through cross-sectional studies (46).

#### 2.4.3 OPN as a potential therapeutic target

OPN not only plays a crucial role in the pathogenesis of NAFLD but is also implicated in a variety of chronic liver diseases (70). OPN is also inextricably linked to various bone diseases, and OPN is a downstream signaling molecule activated by RANK/RANKL that promotes osteoclast differentiation and proliferation (77). OPN deficiency can improve hepatic lipid accumulation and fibrosis and can also abrogate bone resorption. It is tempting to speculate that OPN may provide a potential therapeutic target for the treatment of NAFLD/NASH and its metabolic bone disease-related complications.

### 2.5 TNF-α

TNF-α is produced mainly by activated macrophages, T cells, and natural killer (NK) cells. It is a pro-inflammatory factor that participates in the immune response and plays an important role in controlling infection and protecting the body from invasion. Besides, TNF-α also affects metabolism and aggravates abnormal glucose and lipid metabolism, and it is probable that TNF-α also has adverse effects on bone reconstruction.

#### 2.5.1 Elevated TNF-α levels in patients with NAFLD/NASH

NAFLD is a chronic inflammation of the liver. In NAFLD, the secretion of inflammatory cytokines, including TNF-α by Kupffer cells, NK cells and NKT cells in the liver, increases, and those cytokines are released into the circulation (78). On the one hand, NAFLD further aggravates liver damage, promoting the progression of NAFLD to NASH (79); on the other hand, these inflammatory cytokines released in NAFLD will also cause an inflammatory response or damage in other parts of the body.

#### 2.5.2 TNF-α induces bone loss

Extensive studies have shown that TNF-α is a key molecule that causes osteoclastogenesis and inflammatory bone resorption during inflammatory arthritis. Systemic administration of TNF-α has been shown to promote robust osteoclast formation by directly targeting macrophages in the stromal environment and this occurs only in the presence of permissive level of RANKL (80, 81). TNF-α also enhances M-CSF-induced osteoclastogenesis. In addition, TNF-α suppresses recruitment of osteoblasts from progenitor cells and inhibits osteoblast differentiation (82, 83). It has been demonstrated that estrogen deficiency promoted the production of TNF-α as well, which induced bone loss (84). Given the effect of TNF-α on bone resorption and bone destruction, as well as the inflammatory characteristics of NAFLD, we speculate that TNF-α also plays a role in NAFLD-induced osteoporosis.

#### 2.5.3 Anti-TNF-α treatment

Currently, there are anti TNF-α agents on the market, such as etanercept and infliximab, approved for anti-inflammatory indications. Some studies have found that the increase in OCPs caused by TNF-α can be reversed by anti TNF-α treatment. In addition, other studies have shown that RBP-J, a key upstream negative regulator of osteoclastogenesis, can inhibit excessive bone resorption caused by TNF-α (85). However, whether these reagents can also improve osteoporosis caused by NAFLD remains to be fully investigated.

### 2.6 Fetuin-A

Fetuin-A is a systemic inhibitor of extraosseous calcification that acts as a transporter of calcium and phosphate to promote bone mineralization (86). Fetuin-A is associated with coronary artery disease, ischemic cardiomyopathy, and aortic stenosis and may also function as a positive or negative acute phase protein. Fetuin-A is produced only by the liver, and, in people with fatty liver or hepatitis, fetuin-A is secreted in high concentrations into serum; therefore, fetuin-A may be a useful marker for obesity and fatty liver with insulin resistance.

#### 2.6.1 Elevated serum fetuin-A level in abnormal glycolipid metabolism

Mathews et al. found that, when fed a HFD, fetuin-A knockout mice did not experience significant weight gain and remained insulin sensitive; This suggests that fetuin-A may play an important role in glycolipid metabolism and may be a new therapeutic target for the treatment of type 2 diabetes, obesity, and other insulin-resistant diseases (87). Then, in a cross-sectional study, Stefan et al. found that fetuin-A serum levels were negatively correlated with insulin sensitivity, and fetuin-A serum levels were positively correlated with liver fat in longitudinal studies (88). In another clinical study, adult patients with biopsy-confirmed NAFLD were found to have serum fetuin-A levels that were significantly elevated and positively associated with insulin resistance (89).

#### 2.6.2 Reduced serum fetuin-A levels in patients with osteoporosis

Özkan et al. tested the serum fetuin-A levels of 50 postmenopausal women—25 with osteoporosis and 25 healthy controls—and showed that the serum levels of fetuin-A in the osteoporosis group were generally lower than those in the healthy control group (90).

#### 2.6.3 Decreased fetuin-A levels cause abnormal bone metabolism

A study in fetuin-A-deficient *Ahsg^−/−^* mice reported that long bone growth was impaired, and the growth plates closed prematurely in this background. Fetuin-A mediates the formation of stable colloidal mineral–protein complexes called calciprotein particles (CPPs). Effectively clearing CPPs, thereby removing excess minerals from the circulation, can prevent local accumulation of minerals and calcification of soft tissues. In addition to binding to calcium phosphate, fetuin-A acts as a carrier for lipids, including steroid hormones, which are potent regulators of bone growth and may affect calcification (91). Therefore, if fetuin-A targeting is to be pursued to treat obesity or type 2 diabetes, the potentially deleterious effects fetuin-A on bone homeostasis need to be considered, similar to the case for FGF21 therapy.

#### 2.6.4 Therapeutic potential of fetuin-A

Presently, we are not aware of any drugs based on fetuin-A, which may be because it has such a wide range of physiological activities. High concentrations of fetuin-A can damage pancreatic β cells and cause insulin resistance, but low concentrations of fetuin-A cause osteoporosis and cardiovascular disease by affecting mineralization. In fact, fetuin-A could be considered as a biomarker of NAFLD (92, 93), but how to rationally use fetuin-A for clinical treatment requires further study.

## 3 Conclusion and perspectives

Liver is the center of metabolism, and it is also considered an endocrine organ. In past decades, the crosstalk between liver and heart and between liver and adipose tissue has been widely studied, but the link between liver and bone remains poorly characterized. With the increasing incidence of NAFLD, an improved understanding of the pathophysiological effects of NAFLD that extend beyond the liver is needed to guide patient care. Clinical evidence demonstrates that NAFLD is associated with type 2 diabetes mellitus, cardiovascular disease, chronic kidney disease, and polycystic ovarian syndrome. Currently, there is no consensus regarding whether NAFLD is an independent risk factor for osteoporosis, and the potential role of liver stiffness in osteoporosis development also remains unclear. Additional large prospective studies of well-characterized cohorts of patients with NAFLD are needed to settle the issue of NAFLD as an independent risk factor for osteoporosis.

**Figure 5:**
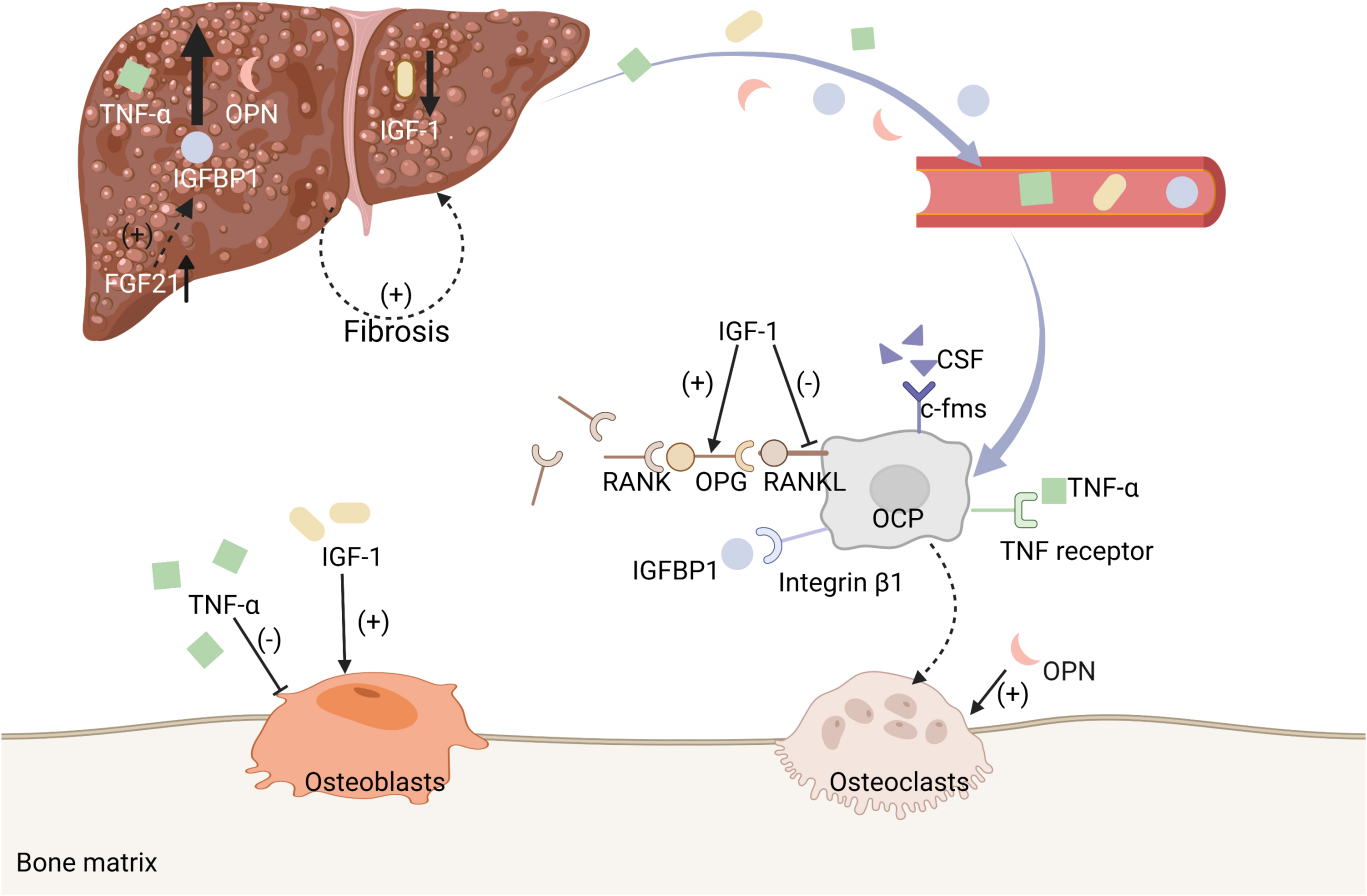
Some molecules secreted by liver are associated with bone metabolism. A summary of mechanisms of some of the enzymes and metabolites secreted by liver that are associated with bone metabolism, including IGF-1, FGF21−IGFBP1, TNF-α, and OPN.

In this review, we discussed several endocrine factors and metabolites secreted by liver and involved in the regulation of bone metabolism, including IGF-1, FGF21, IGFBP1, fetuin-A, TNF-α, and OPN (**Figure 5**). Most are studies performed in rodents, and the clinical implications need to be further assessed, as it has become clear that the crosstalk between liver and bone is much more complicated than currently appreciated. Powerful multi-omics approaches are creating new opportunities to annotate the proteins and metabolites secreted by liver in non-pathological and disease contexts. Mining of these data, together with functional studies and disease modeling, are likely to rapidly advance our understanding of liver−bone crosstalk in the coming years and to inform clinical strategies to manage bone disease in patients with NAFLD.

## Author contributions

XX, JZ and HL, Conceptualization. JZ and XX, Writing - original draft. XX, Writing - review & editing. XX, Supervision and funding acquisition. JZ& TW, Visualization. All authors contributed to the article and approved the submitted version.
